# Insights into the Detoxification of Spruce Monoterpenes by the Eurasian Spruce Bark Beetle

**DOI:** 10.3390/ijms251810209

**Published:** 2024-09-23

**Authors:** Aisha Naseer, Vivek Vikram Singh, Gothandapani Sellamuthu, Jiří Synek, Kanakachari Mogilicherla, Ladislav Kokoska, Amit Roy

**Affiliations:** 1 Faculty of Forestry and Wood Sciences, Czech University of Life Sciences Prague, Kamýcká 129, Praha-Suchdol, 165 00 Prague, Czech Republicsellamuthu@fld.czu.cz (G.S.); synekj@fld.czu.cz (J.S.); mogilicherla@fld.czu.cz (K.M.); 2 Institute of Forest Ecology, Slovak Academy of Sciences, Štúrova 2, 960 53 Zvolen, Slovakia; 3 ICAR-Indian Institute of Rice Research (IIRR), Rajendra Nagar, Hyderabad 500030, Telangana, India; 4 Faculty of Tropical AgriSciences, Czech University of Life Sciences Prague, Kamýcká 129, Praha-Suchdol, 165 00 Prague, Czech Republic; kokoska@ftz.czu.cz

**Keywords:** Norway spruce, monoterpenes, bark beetles, detoxification, RNA-seq, enzyme assay, RT-qPCR, bioassay

## Abstract

Plant defence mechanisms, including physical barriers like toughened bark and chemical defences like allelochemicals, are essential for protecting them against pests. Trees allocate non-structural carbohydrates (NSCs) to produce secondary metabolites like monoterpenes, which increase during biotic stress to fend off pests like the Eurasian spruce bark beetle, ESBB (*Ips typographus*). Despite these defences, the ESBB infests Norway spruce, causing significant ecological damage by exploiting weakened trees and using pheromones for aggregation. However, the mechanism of sensing and resistance towards host allelochemicals in ESBB is poorly understood. We hypothesised that the exposure of ESBB to spruce allelochemicals, especially monoterpenes, leads to an upsurge in the important detoxification genes like P450s, GSTs, UGTs, and transporters, and at the same time, genes responsible for development must be compromised. The current study demonstrates that exposure to monoterpenes like R-limonene and sabiene effectively elevated detoxification enzyme activities. The differential gene expression (DGE) analysis revealed 294 differentially expressed (DE) detoxification genes in response to R-limonene and 426 DE detoxification genes in response to sabiene treatments, with 209 common genes between the treatments. Amongst these, genes from the cytochrome P450 family 4 and 6 genes (CP4 and CP6), esterases, glutathione S-transferases family 1 (GSTT1), UDP-glucuronosyltransferase 2B genes (UDB), and glucose synthesis-related dehydrogenases were highly upregulated. We further validated 19 genes using RT-qPCR. Additionally, we observed similar high expression levels of detoxification genes across different monoterpene treatments, including myrcene and α-pinene, suggesting a conserved detoxification mechanism in ESBB, which demands further investigation. These findings highlight the potential for molecular target-based beetle management strategies targeting these key detoxification genes.

## 1. Introduction

Plants have evolved a diverse array of defence mechanisms to protect themselves against biotic and abiotic stressors. These defences include physical barriers such as toughened bark and resin ducts, as well as chemical deterrents like allelochemicals. Among the carbon reserves of the tree, non-structural carbohydrates (NSCs) play a critical role by providing the nutrient pool required for various physiological processes, including the production of constitutive and induced secondary metabolites. These secondary metabolites, including terpenes and phenolics, are crucial for plant defence. Monoterpenes, C10-compounds composed of two isoprene units, are particularly prominent qualitatively and quantitatively compared to other terpenoids as defence compounds, due to their significant role in deterring herbivores and pathogens [1]. Previous studies have elaborated on a significant increase in the proportion of monoterpenes in response to stressors such as fungal inoculation, drought, and methyl jasmonate (MeJA) infusion, indicating their role in both constitutive and induced defence mechanisms [2,3,4,5].

Among many herbivores that challenge these defences, the Eurasian spruce bark beetle (ESBB, *Ips typographus*) represents one of the most destructive pests in coniferous forests, particularly targeting Norway spruce (*Picea abies* L. Karst.). Despite its sophisticated chemical defence, including producing toxic allelochemicals, *I. typographus* has exploited Norway spruce trees, leading to severe economic and ecological damage to the central European conifer forest [6,7,8]. The host colonisation starts by targeting mature, weakened trees by pioneer males using aggregation pheromones to attract additional beetles. They feed on the phloem, construct mating chambers, and lay eggs, leading to extensive damage as the larvae create radial galleries in the bark. The success of bark beetles in infesting and damaging trees despite their chemical defences is primarily due to their evolved mechanisms for overcoming these defences through the sequestration and detoxification of host allelochemicals [9,10]. Such attacks are also overwhelmed by the microbiome of the bark beetle (together referred to as bark beetle holobiont) that also uses the carbon sources of the host trees (mostly phenols) and metabolises them to semiochemicals or detoxifies them to less toxic forms [11,12,13,14,15,16,17].

*I. typographus* employs a sophisticated detoxification strategy involving several enzymatic systems, which allows it to use plant chemicals for pheromone biosynthesis and detoxification. It utilises the mevalonate and geranyl pyrophosphate (GPP) pathways to convert α-pinene into various pheromones, including 4S-(−)-*cis*-verbenol and 2-methyl-3-buten-2-ol [10,18,19,20]. The detoxification pathways are complex and comprise three main phases: phase I includes lipophilic attacking enzymes such as cytochrome P450 mono-oxygenase (P450), dehydrogenase, peroxidase, hydrolysis enzymes like esterases: carboxycholine (CCE)/acetylcholine esterases (AChE), and esterase families (EST), epoxide hydrolase, and reductase like NADPH-cytochrome P450 reductase (CPR); phase II involves the conjugation of the activated intermediates by enzymes like glutathione S-transferases (GST), UDP-glucuronosyltransferase (UGT), sulfotransferases, N-acetyltransferases, or acyltransferases, rendering them hydrophilic; and lastly, phase III entails the transport of these less toxic forms out of the cells via multidrug resistance proteins [21,22,23,24]. The MRPs are members of ATB-binding cassette transporters (ABC transporters) that cause the ATP-dependent transport of the hydrophobic products of phase I and II to an extracellular medium such that they can be excreted out of the insect body through body fluids [25,26,27,28,29].

Recent reports detail the detoxification, digestion, and defence mechanisms in *I. typographus* and the eight-toothed bark beetle (*Ips sexdentatus*) across different life stages and feeding behaviours [30,31,32]. The key detoxification genes upregulated during feeding or exposure to toxins include cytochrome P450s from families 4, 6, and 9, also found in related beetles like *Dendroctonus* [33,34,35]. Our previous work catalogued detoxification enzymes such as GSTs, UGTs, ABC transporters, and esterases in *I. typographus* [31]. Our lab studies (*unpublished data*) further revealed that beetles feeding on Norway spruce treated with MeJA, which increases toxin levels, exhibit detoxification gene expression compared to those feeding on long-term stored Norway spruce logs. Based on these findings, we hypothesised that the level and type of host chemical exposure directly affect gene expression in *I. typographus*, influencing their survival and successful establishment on the host. Since the allelochemicals in the host bark are present in mixtures, the individual roles and toxicity of monoterpenes in the interaction between Norway spruce and *I. typographus* have not yet been evaluated. In this study, we selectively evaluated the toxicity of five important Norway spruce monoterpenes, *viz*, α-pinene, sabiene, myrcene, R-limonene, and S-limonene against *I. typographus* using the fumigation bioassay [36]. Our results showed that the survival of the beetles decreased inversely with time of exposure in a dose-dependent manner. We examined the effects of the most effective monoterpenes, sabiene, and R-limonene, on gene expression in *I. typographus* using RNA-seq. Notably, exposure to monoterpenes induced the upregulation of a common set of detoxification genes throughout the different chemicals tested and suppressed developmental genes, affirming that specific gene sets are consistently expressed in response to host chemicals and could be potential targets for future pest management strategies for *I. typographus*.

## 2. Results

### 2.1. Toxicity Assay

**Figure 1:**
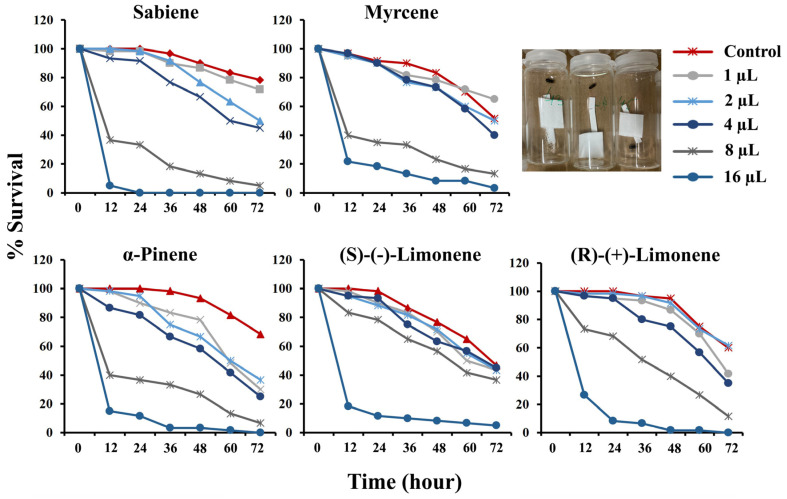
Monoterpene bioassay (via fumigation method). The bark beetle survival distribution curve against the five tested monoterpenes was plotted against a 12 h interval for 72 h against the tested dose (*n* = 60 per dose per chemical). Different colours represent the dose applied in µL per 20 mL of air in the vials.

The toxicity assay was performed for five monoterpenes by fumigating a single beetle for 72 h for each chemical (Table 1). From the sex-specific bioassay, we found no sex-specific effect on the mortality of the male and female beetles after 48 h (*p* > 0.05 in all chemicals). The bioassay revealed that the survival of the beetles is inversely proportional to the time of fumigation in a dose-dependent manner. The mortality increased and was highest at 72 h, with the control mortality surpassing the optimal limit of 15–20% (Figure 1). Hence, the lethal concentration for 70% mortality (LC_70_) was calculated at 48 h. The LC_70_ for the 48 h incubation of *I. typographus* was recorded as *v*/*v* per 20 mL per individual (Table 1, Supplementary File S1). The LC_70_ values at 48 h ranged from ~6 to 10 µL/20 mL. Sabiene was recorded as the most toxic chemical at the lowest LC_70_ of 6.01 µL/20 mL, followed by α-pinene with an LC_70_ of 6.57 µL/20 mL and myrcene with 8.05 µL/20 mL, (R)-(+)-limonene with 8.42 µL/20 mL and the highest LC_70_ of (S)-(−)-limonene at 10.64 µL/20 mL. To see the gene expression difference, we selected the two monoterpenes, R-limonene (moderate LC_70_) and sabiene (lowest LC_70_).

### 2.2. Reference Gene Selection

**Figure 2:**
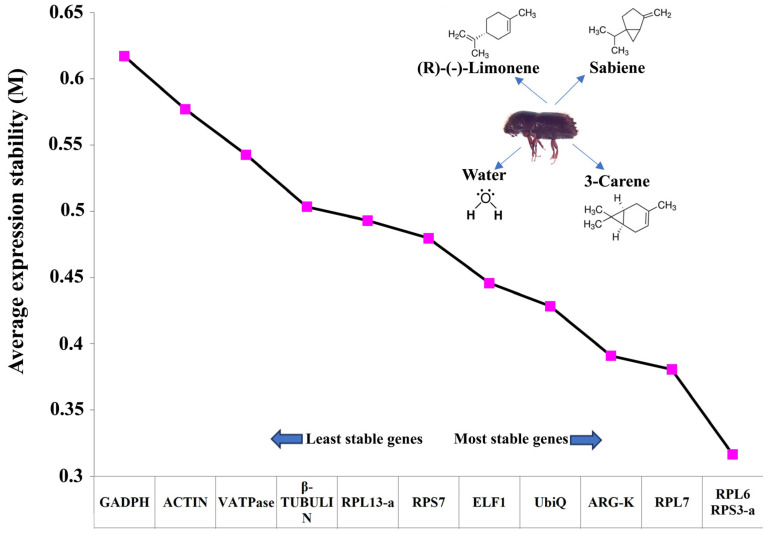
The geNorm comprehensive ranking of the least stable and most stable reference gene combination after monoterpene treatment based on their stability value plotted on the *y*-axis for each of the 12 genes on the *x*-axis. Three individual monoterpene treatments, R-limonene, sabiene, and 3-carene, were tested to select the suitable reference gene. Five different algorithms were used based on the Ct-values generated for each of the 12 housekeeping genes after RT-qPCR (*n* = 4), *viz.*, the ΔCt method, BestKeeper, RefFinder, and NormFinder. The comprehensive ranking was generated using geNorm.

Among the twelve housekeeping genes proposed by Sellamuthu et al. (2022) [38] for the expression normalisation in monoterpene-treated *I. typographus*, the best combination of stable genes was RPS3-a (Ityp04549) and RPL7 (Ityp01351) (Figure 2, Table 2). These two genes have previously been reported by Sellamuthu et al. (2022) [38] as the best reference genes for *I. typographus* study for various tissues and development stages. RPS3a was consistently ranked the best across all five analyses: the ΔCt method, BestKeeper, RefFinder, NormFinder, and geNorm.

### 2.3. Differential Gene Expression Analysis

**Figure 3:**
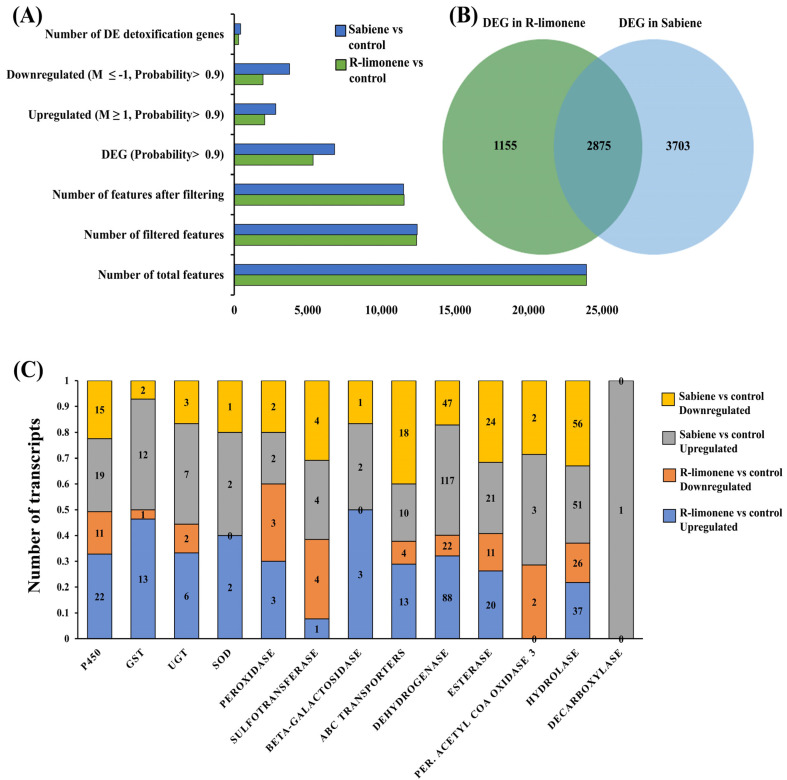
Differential gene expression analysis in fumigated *I. typographus* for the two selected monoterpenes. (**A**) Bar graph representing the number of total genes and DEGs in R-limonene vs. control in green colour and those in sabiene vs. control in blue colour after a cut-off of probability value > 0.9 and log fold change, M ± 1. (**B**) Venn diagram representing the number of common DEGs between the two comparisons. (**C**) Stacked bar graphs comparatively represent the number of expressed detoxification genes in the two treatments. Different colours represent the number of upregulated and downregulated genes in the two chemical treatments, with the individual number of transcripts of each gene family plotted on the *y*-axis. Number inside each bar represents number of transcripts for the corresponding comparison.

From three samples × one biological replicate, a raw library size of approximately 91 million reads was retrieved, which resulted in a 91.8 million normalised library size (Supplementary Files S2 and S3). All the features (reads) generated (Table 3) were mapped back to the reference genome of *I. typographus* [30] to generate the count table. After applying the cut-off for probability >0.9, a total of 5363 and 6815 reads were generated in the R-limonene and sabiene comparison, respectively. These reads/transcripts were further filtered based on M values ± 1 (Figure 3A,B).

#### 2.3.1. R-Limonene Treatment vs. Control

To screen the effects of the R-limonene fumigation treatment on detoxification-related genes in *I. typographus* using DGE, we applied a cut-off probability >0.9 and M value ≥ +1 for upregulation and M ≤ −1 for the downregulation of genes. We reported a total of 2069 upregulated and 1961 downregulated genes, out of which 208 detoxification genes were upregulated and 86 were downregulated. We reported a total of 33 cytochrome P450s, 14 GSTs, 8 UGTs, 2 SODs (superoxide dismutase), 6 peroxidases, 5 sulfotransferases, 3 beta-galactosidase, 17 ABC transporters, 110 dehydrogenases, 31 esterases, 2 peroxisomal acyl-coenzyme A oxidase 3, and 63 hydrolases (Figure 3C, Supplementary Files S3 and S4).

#### 2.3.2. Sabiene Treatment vs. Control

Out of the 2817 upregulated genes (probability > 0.9, and M value ≥ 1) and 3761 downregulated genes (probability > 0.9, and M value ≤ 1), 208 detoxifications were upregulated, and 86 downregulated genes were reported. We reported a total of 24 cytochrome P450s, 11 GSTs, 5 UGTs, 2 SODs, 3 peroxidases, 2 sulfotransferases, 1 beta-galactosidase, 11 ABC transporters, 98 dehydrogenases, 20 esterases, 3 peroxisomal acyl-coenzyme A oxidase 3, and 46 hydrolases. In addition, a single decarboxylase was upregulated (Figure 3C, Supplementary Files S3 and S4).

We recorded more DEGs in the sabiene treatment than in the R-limonene treatment, as also suggested by the lower LC70 value of sabiene compared with R-limonene. In both comparisons, many overexpressed cytochromes belonging to CYP6 and CYP4, the GST sigma class, GSTT1, and UGT B families were found, which are already reported to be mainly associated with detoxification. A large number of dehydrogenases associated with glucose, alcohol metabolism, and aldo-keto conversion were found to be highly upregulated. All the EST family genes were also upregulated. An interesting hydrolase family gene, myrosinase, was reported, which specialises in catalysing the hydrolysis of glucosinolates via the cleavage of thio-linked glucose. Glucosinolates are an important ovipositioning and feeding stimulant in Coleoptera [39].

**Figure 4:**
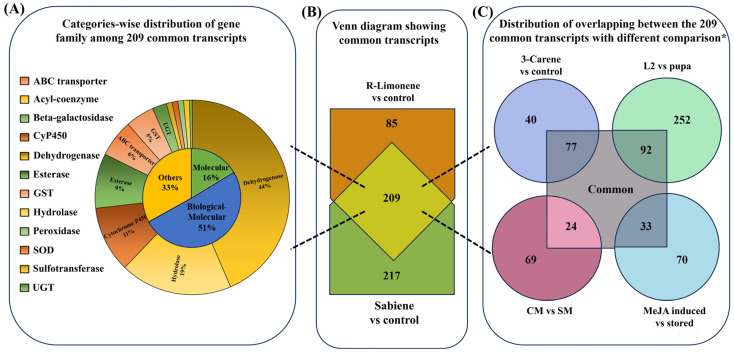
Comprehensive figure representing the distribution of 209 common detoxification genes in *I. typographus* between the two tested monoterpene fumigations. (**B**) Venn diagram showing the common 209 detoxification genes. (**A**) Percentage representation of the detoxification family genes among the 209 common transcripts and the GEO function distribution. (**C**) Comparison of the 209 common genes with four different available transcriptome datasets of *I. typographus* with different treatments: 3-carene vs. control (*unpublished data*); larval stage 2 vs. pupal stage [31]; callow male (CM) vs. sclerotised male (SM) [40]; and *I. typographus* fed on MeJA-treated bark vs. stored bark (Sellamuthu et al., *unpublished data*). * Non-intersecting circles (**C**) do not mean that the comparisons do not have common transcript sequences between them.

**Figure 5:**
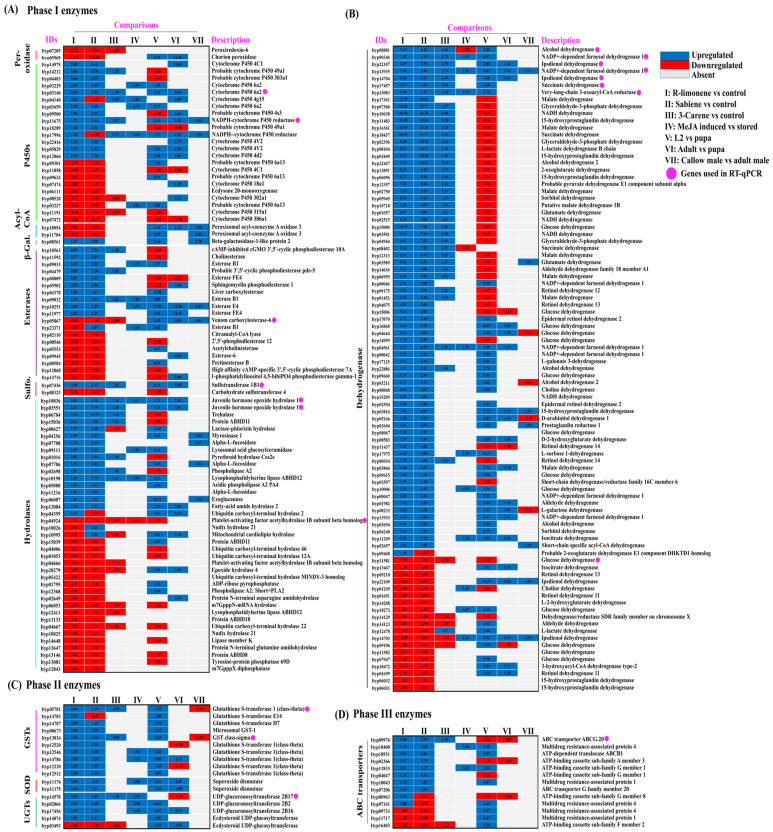
Heat map showing the categorical fold change expression of the 209 common detoxification gene families across the various comparisons used in the study. (**A**,**B**) phase I detoxification enzymes, (**C**) phase II detoxification enzymes, and (**D**) phase III detoxification enzymes. Different comparisons were formulated: (I) R-limonene vs. control, (II) sabiene vs. control, (III) 3-carene vs. control, (IV) MeJA-induced (high) vs. stored (low), (V) L2 vs. pupa, (VI) adult vs. pupa, (VII) callow male vs. sclerotised male. Blue colour represents upregulation, red colour represents downregulation, and white represents that the transcript was either absent or not differentially expressed in the respective comparison (more details in Supplementary File S4). Pink dots mark genes selected for RT-qPCR (refer to Table 5). Expression levels (numbers) for comparisons (I) and (II) were represented based on M value ≥ 1 with probability ≥0.9; the rest of the comparisons (III–VII) were based on logFC ± 1.

In the comparison between the DE genes of R-limonene and sabiene, we found 2875 gene transcripts in common. However, 1155 transcripts were explicitly differentially expressed in the R-limonene comparison, and 3703 were expressed only in the sabiene comparison (Figure 3B, Supplementary File S4). A total of 209 common detoxification genes were found between the two chemical treatments. On comparing these 209 genes with other existing in-house RNA-seq data, we found 77 in common with the genes induced after carene monoterpene treatment (*unpublished data*), 92 in common with genes induced in L2 (the actively feeding stage) compared to ESBB pupa, 33 in common with MeJA-induced spruce log-fed ESBB (*unpublished data*), and 24 in common with the proteome data of the callow vs. sclerotised gut of male ESBBs (Figure 4, Supplementary File S4). Most of them showed the same expression patterns across the two transcriptome comparisons. Over 90% of the genes that occurred in comparison (I) R-limonene and (II) sabiene also have the same expression pattern in carene treatment (III). Similar expressions are also revealed with comparisons like MeJA (IV). However, the expression pattern differs with no external treatment comparisons like the larval–pupal stage (V) and adults (VI) and male guts (VII) (Figure 5). Such expression patterns reveal that a conserved mechanism might be activated when the beetles are exposed to the external overexposure of host allelochemicals or the induced chemical defences of the tree. Interestingly, the expression of genes related to development, such as ecdysone 20-monooxygenase, cytochrome P450 315a1, and chorion peroxidase, was suppressed.

### 2.4. RT-qPCR Analysis and Enzymatic Assay Analysis

**Figure 6:**
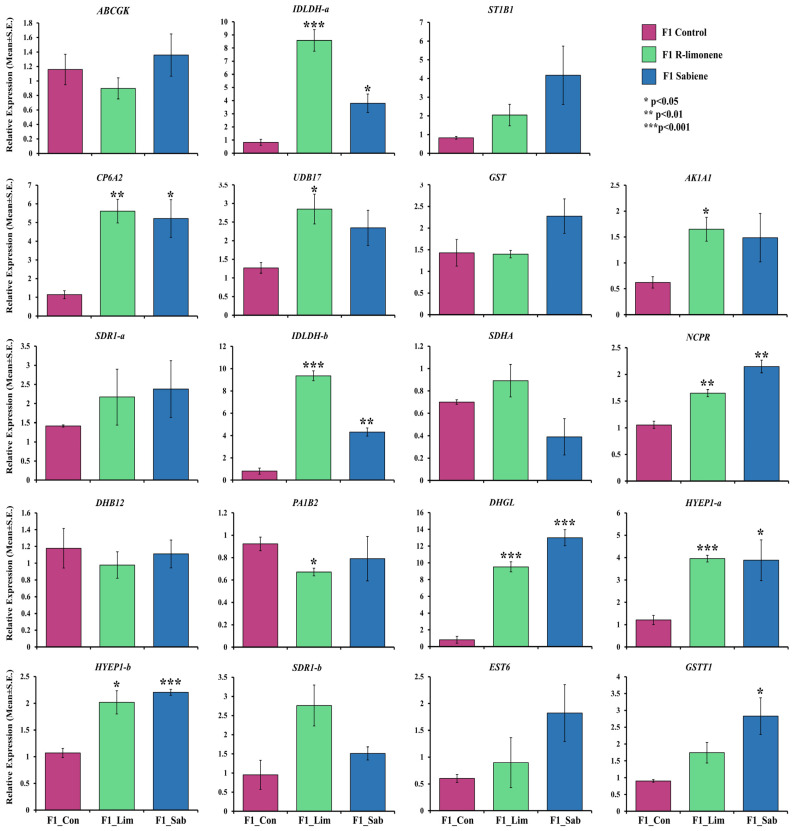
Relative fold change expression (2^(−∆∆Ct)^ ± S.E.) of the 19 important detoxification genes in F1 beetles fumigated with R-limonene and sabiene (*n* = 3). F1 control (pink), F1 R-limonene (green), and F1 sabiene (blue) on the x-axis plotted against their fold change on the y-axis. An independent *t*-test was performed to check the statistical difference between the control and the treatment, and accordingly, *p*-values were generated. * represent *p* < 0.05, ** represent *p* < 0.01, and *** represent *p* < 0.001. *ABCGK*–ABC transporter G family member 20, *IDLDH-a*–Ipsdienol dehydrogenase, *ST1B1*–Sulfotransferase 1B1, *CP6A2*–Cytochrome P450 6a2, *UDB17*–UDP-glucuronosyltransferase 2B17, *GST*–Glutathione S-transferase, *AK1A1*–Alcohol dehydrogenase, *SDR1-a*–Farnesol dehydrogenase, *IDLDH-b*–Ipsdienol dehydrogenase, *SDHA*–Succinate dehydrogenase, *NCPR*–NADPH-cytochrome P450 reductase, *DHB12*–17-beta-hydroxysteroid dehydrogenase 12, *PA1B2*–Platelet-activating factor acetylhydrolase IB subunit beta homolog, *DHGL*–Glucose dehydrogenase, *HYEP1-a*–Juvenile hormone epoxide hydrolase 1, *HYEP1-b*–Juvenile hormone epoxide hydrolase 1, *SDR1-b*–Farnesol dehydrogenase, *EST6*–Venom carboxylesterase-6, and *GSTT1*–Glutathione S-transferase 1.

**Figure 7:**
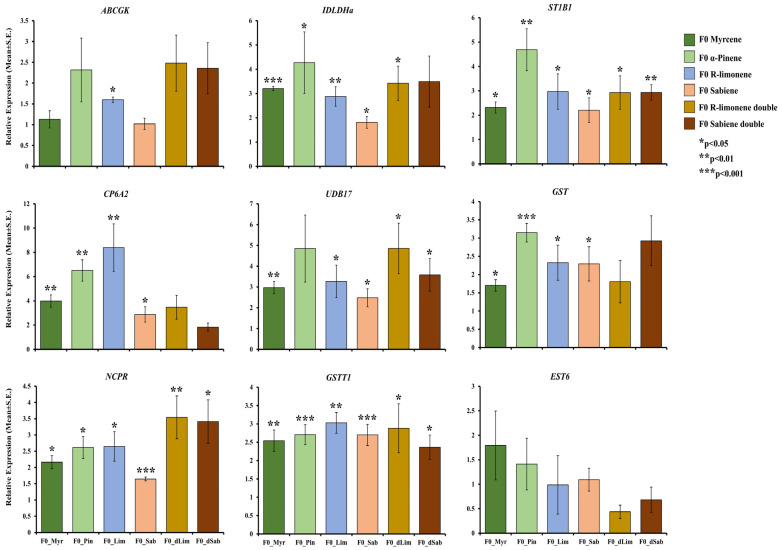
Relative fold change expression (2^(−∆∆Ct)^ ± S.E.) of the selected 9 important detoxification genes in F0 beetles fumigated with myrcene (dark green), α-pinene (green), R-limonene (blue), and sabiene (orange) at LC_70_-48 h (*n* = 4); and F0 beetles treated with a double dose of LC_70_ of R-limonene (yellow) and sabiene (brown) each (*n* = 5) for 48 h on the *x*-axis plotted against their fold change on the *y*-axis. An independent *t*-test was performed to check the statistical difference between the control and the treatment, and accordingly, *p*-values were generated. * represent *p* < 0.05, ** represent *p* < 0.01, and *** represent *p* < 0.001. *ABCGK*–ABC transporter G family member 20, *IDLDH-a*–Ipsdienol dehydrogenase, *ST1B1*–Sulfotransferase 1B1, *CP6A2*–Cytochrome P450 6a2, *UDB17*–UDP-glucuronosyltransferase 2B17, *GST*–Glutathione S-transferase, *AK1A1*–Alcohol dehydrogenase, *SDR1-a*–Farnesol dehydrogenase, *IDLDH-b*–Ipsdienol dehydrogenase, *SDHA*–Succinate dehydrogenase, *NCPR*–NADPH-cytochrome P450 reductase, *DHB12*–17-beta-hydroxysteroid dehydrogenase 12, *PA1B2*–Platelet-activating factor acetylhydrolase IB subunit beta homolog, *DHGL*–Glucose dehydrogenase, *HYEP1-a*–Juvenile hormone epoxide hydrolase 1, *HYEP1-b*–Juvenile hormone epoxide hydrolase 1, *SDR1-b*–Farnesol dehydrogenase, *EST6*–Venom carboxylesterase-6, and *GSTT1*–Glutathione S-transferase 1.

To validate the RNA-seq-based gene expression data, we performed an RT-qPCR analysis on F1 beetles treated with R-limonene and sabiene for 19 selected detoxification genes. These included cytochrome P450, GST, esterase, ABC transporter, juvenile hormone epoxide hydrolase, and dehydrogenases. The RT-qPCR expression showed a similar expression pattern of these genes as the RNA-seq data (Figure 6, Supplementary File S5). Most of the genes had a significantly high expression fold change. Further, to assess the effect of generation and conserved upregulated detoxification genes on monoterpene overexposure, we tested some of these genes with an RT-qPCR on the F0 beetle population treated with R-limonene, sabiene, myrcene, and α-pinene (Figure 7). The expression in α-pinene was the highest throughout all the nine tested genes, followed by myrcene, which can be attributed to the involvement of these chemicals in sequestration and pheromone biosynthesis pathways. The mortality assay of F0 beetles with an LC70 dose showed a lower mortality (~30% only) than the expected percentage observed for F1 beetles. Such a lower mortality can be attributed to the higher vigour of the F0 population due to their already-induced detoxification genes. However, with a double LC70 dose, the mortality increased to up to 80% (Supplementary File S1). When the fumigation dose was doubled, the gene expression was higher in the double LC70 dose-treated beetles than the beetles treated at just LC70 in the F0 and F1 populations. These findings assert that the F0 beetles possess a higher vigour, likely due to a higher resistance, associated with a higher expression of detoxification genes.

**Figure 8:**
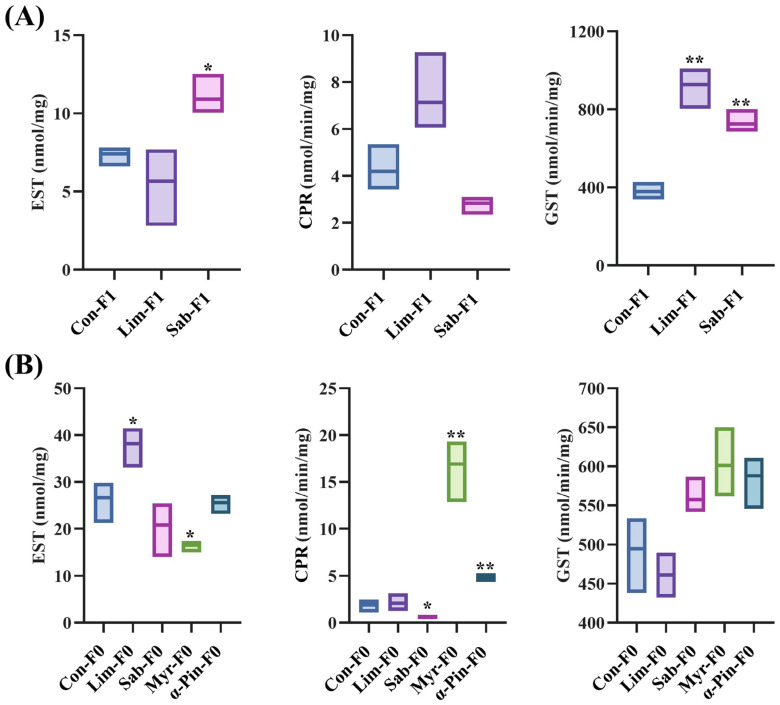
The activities of esterase (EST), cytochrome P450 reductase (CPR), and glutathione S-transferase (GST) measured on (**A**) F1 population of *I. typographus* fumigated with R-limonene and sabiene and (**B**) wild beetle (F0) population fumigated with R-limonene, sabiene, and additionally with myrcene and α-pinene (*n* = 3). An independent *t*-test was performed to check the statistical difference between the control and the treatment, and accordingly, *p*-values were generated. Esterase enzyme activity is expressed as nmol/mg, and that of CPR and GST is expressed as nmol/min/mg. * represent *p* < 0.05, and ** represent *p* < 0.01. F0–wild beetles, F1–first lab-reared generation, Con–control, Lim-(R)-limonene, Sab–sabiene, Myr–myrcene, and α-Pin–α-pinene.

Enzymatic activity assays on CPR, GST, and EST reflected the same expression pattern as the RNA-seq and RT-qPCR, showing the downregulation of esterases and the upregulation of GST and CPR (Figure 8).

## 3. Discussion

The detoxification of spruce monoterpenes by *Ips typographus* plays a pivotal role in the beetle’s ability to colonise and damage spruce forests, leading to significant ecological and economic impacts in Central Europe [6,8]. The beetle’s success in thriving despite exposure to these toxic allelochemicals highlights its highly specialised adaptation involving enzymatic systems that degrade or transform monoterpenes into less harmful forms [41,42]. Often, the divergent response of beetle holobionts against host monoterpenes may determine their niche partitioning strategies. Interestingly, the expression of the gene in specific monoterpenes has not been explored. This study provides new insights into the specific molecular mechanisms that enable *I. typographus* to metabolise toxic monoterpenes such as α-pinene, sabiene, myrcene, and R-limonene, potent defensive allelochemicals produced by spruce trees. The fumigation bioassay conducted in this study revealed that these monoterpenes exhibit significant toxicity to *I. typographus*, with sabiene and R-limonene emerging as particularly effective inhibitors. 

Previous research has shown the induction of defence priming and the elicitation of the induced defense system of the tree during biotic and abiotic stresses [43,44,45,46]. Although some monoterpenes, such as α-pinene and myrcene, have been found to have attractant properties in other beetle species [47,48], they can exhibit inhibitory effects at higher concentrations [49]. Additionally, R-limonene has been reported to cause a higher mortality in other beetle species than α-pinene [33], while its effectiveness varies in different contexts [36]. The direct toxicity of sabiene to bark beetles remains less explored, although its increased production in response to MeJA and fungal inoculation is noted [50]. In this study, the fumigation assays revealed significant differences in the effectiveness of the five monoterpenes tested, with R-limonene and sabiene showing the highest potency. The lower LC_70_ value for sabiene indicates its high effectiveness as a fumigant, whereas R-limonene, although moderately toxic, may require higher concentrations or alternative formulation strategies for optimal pest control (Figure 1, Table 1). Similarly, Chiu et al. (2017) [36] reported that R-limonene exhibits the highest toxicity when used via fumigation against the mountain pine beetle. However, *I. typographus* has developed sophisticated detoxification strategies to overcome these chemical defences, as demonstrated by the changes in gene expression and enzyme activity in response to monoterpene exposure. Previous studies have identified detoxification-related gene families in *I. typographus* at both the RNA and protein levels, showing tissue-specific and feeding-specific behaviour induced by feeding on host tissues [30,31,40]. 

This study, encompassing a gene expression analysis using RNA-seq, RT-qPCR, and enzymatic assays, indicates that the evolution of the detoxification pathways in *I. typographus* is driven by intense selective pressures from the spruce host defence chemistry. The upregulation of detoxification enzymes in response to monoterpene exposure reflects a dynamic, inducible defence mechanism that allows the beetle to rapidly adapt to fluctuating levels of these toxic compounds based on the physiology of the host [51,52]. The involvement of key detoxification enzymes highlights the beetle’s sophisticated metabolic activity tailored to overcoming the host’s chemical defences [32,52,53]. The redundancy and robustness observed in the beetle’s detoxification pathways suggest a high level of metabolic flexibility, allowing *I. typographus* to survive across diverse environmental conditions and variations in host tree chemotypes [32,54,55].

### 3.1. Expression of Phase I Detoxification Enzymes after Monoterpene Exposure

**Figure 9:**
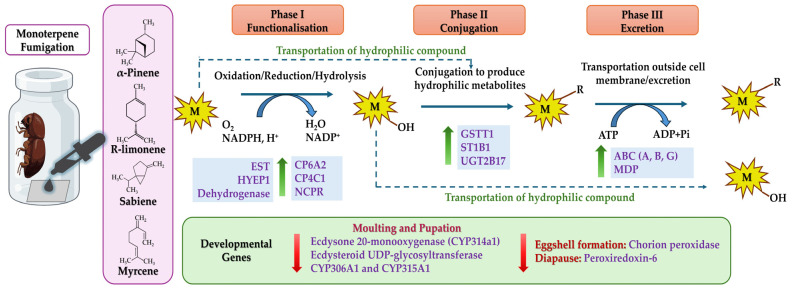
Summary of the key findings in the RNA-seq analysis and RT-qPCR validation of important genes. Monoterpenes (M) are applied to filter paper, and beetles are fumigated for 48 h. M passes through phase I and produces a reactive intermediate (M-OH) after oxidation, reduction, or hydroxylation of the lipophilic compound (M), or it passes directly to phase II. M-OH get conjugated with reduced glutathione or UDP-glucose in phase II to produce a hydrophilic compound (M-R). This hydrophilic compound is transported through the cell membrane into body fluid and then excreted out of the insect body in phase III. Key detoxification genes reported to be upregulated in this study in each phase are denoted by green up-head arrows. Simultaneously, some development-related genes were downregulated, denoted by red down-head arrows. *EST*–esterases, *HYEP1*–juvenile hormone epoxide 1, *CP6A2*–Cytochrome P450 6a2, *CP4C1*–Cytochrome P450 4c1, *NCPR*–NADPH-cytochrome P450 reductase, *GSTT1*–Glutathione S-transferase 1, *ST1B1*–Sulfotransferase 1B1, *UDB17*–UDP-glucuronosyltransferase 2B17, *ABC*–ABC transporter family members, *MDP*–Multidrug resistance-associated protein.

CYP4, CYP6, and CYP9 are the most important detoxification groups reported in other insect species such as *Dendroctonus* [34,35,56,57], *Sitophilus zeamais* [58,59], and *Aphis gossypii* [60]. Dai et al. (2015, 2021) [33,52] also reported several CYPs being expressed due to monoterpene exposure and feeding, and the molecular characterisation of one of the CYP6 genes denotes that their expression is regulated by juvenile hormone (JH) levels. Our study revealed that the most common cytochromes belong to families 4 and 6, the most abundant being CYP6A2. We also report higher levels of JH-related hormones like juvenile hormone epoxide hydrolase 1, a pheromone biosynthesis pathway gene [10,61,62] across chemical treatment comparisons I to III (Figure 5). The most highly expressed cytochrome reported is CYP4C1, which is supposedly involved in breaking down synthetic insecticides and providing cold tolerance and heat resistance in *Bemisia tabaci* [63]. CYP49A1 was overexpressed in three chemical exposures in our study (Figure 5, comparisons I, II, and III). This gene was previously reported in the *D. melanogaster* hindgut of the larval stage and was only overexpressed during the feeding larval stage and not the wandering larval stage [64]. However, Naseer et al. (2023) [31] reported that in *I. typographus*, CYP49A1 was downregulated in the L2 stage, which is a high feeding stage. In our data, the most abundantly upregulated gene across most comparisons was CYP6A2, which is associated with insecticide metabolism, mainly DDT in *Drosophila* and imidacloprid in *Aphidius gifuensis* [65,66,67]. Recently, Tsuji et al. (2024) [68] reported a higher accumulation of CYP6A2 in the gut and salivary glands of *Drosophila* larvae after sesamin feeding. Apart from R-limonene and sabiene, CYP6A2 was also upregulated in the L2 and adult feeding stages, as previously reported (Figure 5A) [31]. Interestingly, in our report, the two most downregulated CYPs were the Halloween genes CYP306A1 and CYP315A1. The expressions of CYP306A1 and CYP315A1 were two times lesser in the R-limonene treatment and four times lesser in sabiene treatment when compared with the pupal stage of ESBB (Figure 5A). CYP306A1 has been previously reported to take part in insect moulting and ecdysone biosynthesis in flies and a lepidopteran insect, *Chilo suppressalis* [69,70]. Another gene, ecdysone 20-monooxygenase, also known as CYP314A1, was also downregulated in our data (Figure 5A, Supplementary File S3), revealing that at a higher toxin exposure, the expression of these developmental genes is lower, resulting in hindered moulting or insect development (Figure 9). We also report that peroxiredoxin-6, reported earlier to be associated with diapause induction in locusts [71], is downregulated in all the chemical treatments. A chorion peroxidase was downregulated in R-limonene and sabiene treatment. Chorion peroxidase is linked with eggshell formation and has been reported to be highly expressed in adult (sclerotised) *I. typographus,* which can start oviposition [31,71].

### 3.2. Expression of Phase II Detoxification Enzymes after Monoterpene Exposure

Glutathione S-transferases (GSTs) facilitate the metabolism of the endo- or exogenous oxidative stress molecules (e.g., insecticides) by reducing them via dehydrochlorination or by conjugating them with reduced glutathione to produce hydrophilic metabolites that can be excreted [72,73]. In our study, we reported 10 GSTs, which are upregulated in both the chemical treatments, denoting their involvement in endogenously catalysing the intermediates produced during the detoxification mechanism and facilitating their solubility and excretion out of the body. Most of them belong to the theta class of GST (Figure 5C). Gao et al. (2020) [74] reported 16 full-length GSTs belonging to class delta, epsilon, theta, and sigma in Chinese pine beetles. GSTT1 possesses peroxidase activities and is also involved in protein binding for organophosphates [75]. The expression of GSTs and esterases are putatively dependent on the host tree chemical composition and have been reported to significantly change with the alteration of the host via diet switch in *I. sexdentatus* [32]. The consistently high significant expression of GSTT1 throughout all the tested monoterpenes in both the F1 and F0 populations in the RT-qPCR reflects the importance of this gene in monoterpene detoxification (Figure 6 and Figure 7), which can be functionally validated in the future. Another important gene consistently upregulated in the RNA-seq and RT-qPCR was sulfotransferase 1B1 (ST1B1), which is reported to have specialised xenobiotic substrate response sites and an involvement in resistance against xenobiotic stress [76].

Insects have evolved to utilise UGTs to glycosylate endogenous or exogenous lipophilic compounds by conjugating them to UDP-glucose as an activated sugar donor, facilitating detoxification [77,78]. In the recent past, UGTs have been established as one of the important genes for metabolising dietary toxins and insecticides in many crop pests like silkworms (*Bombyx mori*), the fall armyworm (*Spodoptera frugiperda*), cotton aphid (*Aphis gossypii*), brown planthopper (*Nilaparvata lugens*), cotton bollworm (*Helicoverpa armigera*), and peach-potato aphid (*Myzus persicae*), to name some [79,80,81,82,83,84,85]. Also, in sucking insects like the Asiatic honeybee, *Apis cerana cerana*, the UGT2B20-like gene plays an important role in pesticide resistance [86]. UGT 2B17 was the most upregulated gene of treatment comparisons I–II (Figure 5C). Li et al. (2017) [87] functionally characterised UGT 2B17 in *Plutella xylostella* using RNA interference (RNAi). Following the knockdown of UGT 2B17, the sensitivity of third-instar larvae to chlorantraniliprole increased by 27.4% in the susceptible population and by 29.8% in the resistant populations. In this study, the RT-qPCR expression of UGT 2B17 was also reported to be significantly high through the different chemical treatments and with the respective generations (Figure 6 and Figure 7). Alternatively, we reported the constant downregulation of ecdysteroid UDP-glycosyltransferase (EGT) in the three chemical treatments (Figure 5C). EGT is a baculovirus-encoded protein, transferred to initially lepidopteran insects via horizontal gene transfer, which inactivates the ecdysone hormone formation in the host insects, thus preventing moulting and pupation [88,89]. Naseer et al. (2023) [31] showed that this gene was highly upregulated during the pupal stage compared to larvae or adults. The downregulation of EGT after the overexposure of allelochemicals in adult beetles shows the conserved nature of insects to save their energy during oxidative stress rather than reproduce. Hence, we can re-infer that exogenous overexposure to plant defence chemicals hinders developmental and reproductive genes and promotes the overexpression of conserved detoxification genes (Figure 9). Two superoxide dismutase (SOD) genes were upregulated in the R-limonene and sabiene comparison (Figure 5C). These genes are involved in tolerance against oxidative stresses [90].

### 3.3. Expression of Phase III Detoxification Enzyme after Monoterpene Exposure

Phase III detoxification enzymes play a crucial role in detoxification by facilitating the removal of xenobiotics and endogenous toxins. ABC transporter genes help the hydrophilic, less toxic metabolised product to be excreted from the body of the insects with body fluid. Previously, Naseer et al. (2023) [31] reported that the expression of these transported genes drastically increases at the second-instar larva stage compared to the pupal stage of *I. typographus*. The pupae are in the non-feeding sedentary stage, where the exposure of the host chemicals is close to none, and only moulting and development occur. The beetle development requires energy derived from already metabolised host tissues ingested during feeding. Hence, the detoxification genes are downregulated during the pupal stages. However, during the callow stage, beetles start to feed again, and the transportation and excretion via body fluid continue. Instead, they must increase the supply of nutrition to keep it circulating for moulting into sclerotised adults. In our study, we reported the ABC transporter being highly upregulated during the vigorous feeding stage of the beetle (Figure 5D). Sun et al. (2017) [91] identified 40 ABC transporters from the ABCA–ABCH subfamilies in *Laodelphax striatellus*, with over 20% of these genes significantly upregulated in resistant strains. Eight genes from the ABCB, ABCC, ABCD, and ABCG subfamilies were consistently upregulated across all resistant strains compared to the susceptible strain. Knocking down genes encoding ABC transporters, either individually or simultaneously, confirmed their role in resistance [92,93,94,95]. We assume that overexposure to the monoterpene leads to a hastened detoxification process, and hence, the need to eliminate the toxins from the beetle body increases, which can be achieved by the overexpression of ABC transporter genes.

## 4. Materials and Methods

### 4.1. Insect Collection and Rearing

Freshly infested Norway spruce logs were collected from research plots managed by the School Forest Enterprise (ŠLP) near Kostelec nad Černými lesy (49.9940° N, 14.8592° E) in the eastern district of the Central Bohemian region of Prague, Czech Republic. During the summer, the area’s climate is drier and warmer, with a growing season of 150 to 160 days, an average annual temperature of 7 to 7.5 °C, and a mean annual precipitation of 600 mm [96,97]. During the summer season, the climatic conditions are suitable for the progeny development and swarming of beetles. The infested trees were cut down, and their logs were then transported to a rearing facility at the Faculty of Forestry and Wood Sciences of the Czech University of Life Sciences, where they were stored at 4 °C until used. Logs were stored for a maximum of two weeks from the date of felling. Beetles were collected from the wild logs and reared on fresh, uninfested spruce logs to produce the F1 generation population. The logs were placed in mesh cages within a laboratory environment where conditions were carefully controlled: a temperature of 25 °C, a relative humidity maintained at 65%, and ample air supplied [31,38]. Emerging F1 beetles were collected, sexed based on pronotum hair and knob dimorphism, weighed, and used on the same day, and those in compromised physiological conditions were excluded from the bioassay.

### 4.2. Fumigation Bioassay and Toxicity Calculation

For the toxicity assays, five monoterpenes (Table 4) were used individually for fumigation assays based on previous reports [3,44,45,98,99] following the protocol of Chiu et al. (2017) [36]. A 1.5 cm × 1.5 cm piece of Whatman filter paper was placed in a 20 mL scintillation vial, onto which defined volumes of undiluted monoterpenes were applied using a pipette (Eppendorf) immediately before adding a single beetle to the vial. A moist filter paper was placed in each vial to maintain humidity, and the vial was closed with a cap and sealed with parafilm. Only moist filter paper was placed in the scintillation vial for the control, a beetle was inserted, and the vial was sealed without any monoterpene application. Monoterpenes were tested at five defined doses (volume monoterpene applied/volume airspace of the assay vial) of 50 μL/L, 100 μL/L, 200 μL/L, 400 μL/L, and 800 μL/L. To achieve the doses of monoterpenes, undiluted monoterpenes were applied at volumes of 1 μL, 2 μL, 4 μL, 8 μL, and 16 μL per 20 mL of the vial, respectively. The set-up was placed inside the climate chamber (Memmert HPP2200ECO, Schwabach, Germany), which maintained a temperature of 25 °C and a 20 h/4 h light/dark photoperiod [100]. The beetles were exposed to volatiles for the pre-optimised time duration of 72 h, and mortality was assessed at every 12 h interval. Beetles were considered dead if they did not show any movement while the vial was being agitated. In total, 360 beetles were tested for each of the monoterpenes. At each chemical dose and control for each monoterpene, 60 insects with 30 females and 30 males were used (*n* = 60/dose/monoterpene). The beetles were distributed so that their average body weight was almost the same for all concentrations. Trials were conducted on each monoterpene in multiple technical replicates due to uneven numbers of beetles collected each day; however, the beetles collected on each day were randomly sorted into control and treatments (for all doses) in equal numbers to minimise the sample distribution biases. There was no significant difference between the body weight of the males and females used for the individual chemical treatment (Supplementary File S1).

To calculate the LC_70_, 48 h mortality data was used, and dose–response analyses were conducted. Out of all the monoterpenes used in the bioassay, the top two most suitable chemicals (one with the lowest LC_70_ and another with a moderate LC_70_) were used for the RNA-seq. For the RNA-seq, freshly emerging F1 *I. typographus* were treated individually again at LC_70_ with R-limonene and sabiene for 48 h; then, the collected beetles were snap-frozen in liquid nitrogen and stored at −80 °C. A total of three treated beetles were pooled together to make one biological replicate, and their whole body was crushed using a pre-chilled mortar and pestle. The crushed beetle samples were stored at −80 °C for further RNA extraction. For the RT-qPCR, the bioassay was repeated with new F1 and F0 ESBB populations and four chemicals (R-limonene, sabiene, α-pinene, and myrcene) at their respective LC_70_ for 48 h.

To check the effect on beetle generation, a double dose of the LC_70_ was applied to F0 beetles for R-limonene and sabiene. For the RT-qPCR and enzyme assays, RNA was extracted from the raised samples after 4 beetles were pooled together to make one biological sample for the LC70, and 2 beetles were pooled for the double LC_70_. 

### 4.3. Total RNA Extraction, cDNA Synthesis, and RT-qPCR Analysis

Total RNA was isolated using the PureLink™ RNA Kit from Ambion (Invitrogen, Carlsbad, CA, USA) following the manufacturer’s protocol. The isolated RNA was treated with DNase I (TURBO DNase Kit, Ambion, Austin, TX, USA). One µg of RNA was used to synthesise cDNA using a High-Capacity cDNA Reverse Transcription Kit (Applied Biosystems Life Technologies, Waltham, MA, USA) and stored at −20 °C until further use. The cDNA was diluted 5-fold to be used as a template for RT-qPCR. Three biological replicates were used for F1 beetles, four biological replicates per treatment were used for F0 beetles at LC_70_, and five biological replicates were used for the double LC_70_ for F0 beetles for RT-qPCR. The primers were designed using IDT PrimerQuest software (IDT, Leuven, Belgium) (Table 5). The RT-qPCR was performed as reported by Naseer et al. (2023) [31]. Briefly, a 10 µL reaction mixture was prepared using 5 µL of 2x SYBR^®^ Green PCR Master Mix (Applied Biosystems, Waltham, MA, USA), 3 µL RNase-free water (Invitrogen, Waltham, MA, USA), 1.0 µL of cDNA, and 0.5 µL each of 10 µM forward and reverse primers. The Applied Biosystems™ StepOne™ Real-Time PCR System (Applied Biosystems) was set up with the following reaction conditions: initial denaturation at 95 °C for 10 min, followed by 35 cycles of 95 °C for 15 s, 60 °C for 1 min, and a dissociation curve analysis during which temperature was increased from 60 to 95 °C. The 2^(−ΔΔCt)^ method [101] was used to calculate the relative expression levels of the target genes. Ribosomal protein L7 (RPL7) served as a reference gene for expression normalisation [31,38].

### 4.4. Reference Gene Selection

Twelve candidate reference genes for the fumigation of ESBB with (R)-(+)-limonene, sabiene, and 3-carene were selected based on a previous study (Table 2) [38]. Four different programs (NormFinder, ΔCt, BestKeeper, and RefFinder) were used to check the expression stability of the candidate genes. The genes were ranked according to their overall performance in three chemical treatments and a control using geNorm. Four biological replicates were used for each treatment and control.

### 4.5. RNAseq Analyses

A pairwise differential expression analysis (without replicates) was performed using OmicsBox (version 3.2.2) [102], using the software package NOISeq [103,104], in a nonparametric approach. The contrasting log fold change difference (M) and absolute expression difference (D) between the test (chemical treatment-R-limonene and sabiene) and reference (control) were ascertained. A single replicate was fed into the NOISeq pipeline, and five technical replicates were simulated for each experiment condition, assuming that the read counts followed the multinomial distribution. Preprocessing of the raw read was performed using the following criteria: CPM—1.0, normalisation method—TMM (trimmed mean of M values), number of simulated replicates—5, size of simulated replicates—0.2, and variability—0.02. For the differential expression analysis, the probability value > 0.9 was chosen. A gene set enrichment analysis (GSEA) was performed using the ranking generated using the formula: −sign(M)∗sqrt(M^2^ + D^2^) (Supplementary File S2).

### 4.6. Comparison between Multiple In-House Data

To compare the differential gene expression data of the two chemicals and identify the vital detoxification gene across the two comparisons (I: R-limonene vs. control and II: sabiene vs. control), and in-house carene-fumigated beetles vs. control (III) (*unpublished data*), ESBB feeding on MeJA-treated bark vs. those feeding on stored bark (IV) (*unpublished data*), active feeding stage vs. non-feeding stage (L2-larval stage two vs. T4-pupa (VI) and adult vs. pupa stage (VI)) data of ESBB from Naseer et al. (2023) [31], and callow male vs. sclerotised male (VII) (CM vs. SM) data from Ashraf et al. (2023) [40] were used (Supplementary File S4). The cut-off used for each of the comparisons (III–VII) was FDR *p* < 0.05 and log fold change ± 1 (M value in case of I and II). The fold change value of the common gene transcripts between I and II, among all other comparisons after applying the cut-off, was retrieved and plotted in the heat map (Figure 5).

### 4.7. Enzyme Activity Assay

An enzyme activity assay was performed for three genes, glutathione S-transferase (GST), cytochrome P450 reductase (CPR) and esterase (EST), to comparatively assess the enzyme activity in *I. typographus* treated with four different chemicals following the preoptimised protocols of Naseer et al. (2023) and Sellamuthu et al. (2024) [31,32]. The enzyme activities were assessed following the published protocol with minor modifications [105]. Briefly, the beetle whole body powder was homogenised in 50 mM of Na_3_PO_4_ (pH 7) containing 1 mM EDTA and 0.1 mM DTT. The solution was centrifuged, and the supernatant containing total protein was collected as an enzyme source. The protein concentration was measured using the Bradford method [106] with BSA and used to correct the enzyme activities as standard.

### 4.8. Statistical Analysis

The percentage mortality of the beetles was calculated using Henderson–Tilton’s formula [37]. The dose–response analysis was performed using XLSTAT 2020 (v 3.1.1011) to calculate the LC_70_. An F-test and Student’s *t*-test were performed to check the sex-based mortality among the populations (Supplementary File S1). To compare the relative expression of detoxification genes between the control and treated beetles in the RT-qPCR and enzyme activity assay, first, the normality of each group was checked using the Shapiro–Wilk test, and then the variance homogeneity was calculated between the control and treatment groups using Levene’s test. Then, an independent *t*-test was performed with an equal variance Student’s *t*-test (if Levene’s *p* > 0.05) or unequal variance Welch’s *t*-test (if Levene’s *p* < 0.05) accordingly, and *p*-values were generated based on the significant differences between control and treatment groups at 95 C.I. using RStudio (version 4.2.3) (Supplementary File S5). The statistical and numerical analysis used in the DGE data is described in Section 4.5 under ‘RNA-seq analyses’.

## 5. Study Limitations

The RNA study is conducted based on a single replicate (n − 1) and may have some biases. We used an RT-qPCR and other in-house transcriptome data to gain a higher reliability for and insight into our current findings. Though the RT-qPCR data show considerable correspondence to the transcriptome data, we recommend including more biological replicates to enhance the robustness of the RNA-seq analysis.

## 6. Conclusions

This study extends our understanding of the detoxification mechanisms of allelochemicals in *I. typographus* by investigating the toxicity of five key monoterpenes (α-pinene, sabiene, myrcene, R-limonene, and S-limonene) found in Norway spruce bark. With the integration of mortality assays and chemical treatments, our findings demonstrate a dose-dependent decrease in beetle survival upon exposure to these monoterpenes, with sabiene and R-limonene proving particularly effective. The RNA-seq analysis revealed a significant upregulation of detoxification genes and suppression of developmental genes in response to these potent monoterpenes. Furthermore, the RT-qPCR and enzymatic assays corroborated the RNA-seq results, suggesting the conserved regulation of detoxification pathways triggered by exogenous host allelochemicals that demands further experimental corroboration. In conclusion, this study deepens our understanding of monoterpene toxicity in forest beetles, and the integration of bioassay and molecular data provides a solid foundation for future bark beetle adaptation research, illuminating the complex interactions between monoterpenes and beetle physiology. These insights pave the way for developing innovative target gene-based management strategies (i.e., RNA interference) for a more effective and sustainable control of bark beetles, including *I. typographus*, addressing critical needs in forest pest management and promoting the advancement of targeted pest control solutions [107,108].

## Figures and Tables

**Table 1:** Toxicity assay. Monoterpenes and their respective LC_50_ and LC_70_ values against *Ips typographus*.

Chemical	48 H			%Corrected Beetle Mortality for Different Doses *				
	LC_50_ (µL/20 mL)	LC_70_ (µL/20 mL)	% Control Mortality	1 (µL/20 mL)	2 (µL/20 mL)	4 (µL/20 mL)	8 (µL/20 mL)	16 (µL/20 mL)
Sabiene	4.4	6.0	10.0	−4.0	8.0	20.0	84.0	100.0
α-Pinene	3.6	6.6	6.7	16.1	28.6	37.5	71.4	96.4
Myrcene	5.3	8.1	16.7	6.0	12.0	12.0	72.0	90.0
(R)-(+)-Limonene	6.2	8.4	5.0	8.8	3.5	21.1	57.9	98.3
(S)-(−)-Limonene	7.3	10.6	23.0	8.7	6.5	17.4	26.1	89.1

* *The percentage corrected mortality was calculated for each dose using Henderson–Tilton’s formula* [37].

**Table 2:** Reference gene identification. Ranking of the 12 candidate reference genes based on their stability values performed by ∆Ct, BestKeeper, RefFinder, and NormFinder after fumigation with three monoterpenes.

Sr. No.	Genes	ΔCt Method		BestKeeper		RefFinder		NormFinder		Recommended Genes
		Stability	Rank	Stability	Rank	Stability	Rank	Stability	Rank	
1	RPS3-a	0.52	1	0.31	2	1.19	1	0.144	1	**RPS3-a** and **RPL7**
2	Arg-K	0.54	2	0.38	6	3.25	4	0.152	2	
3	RPL7	0.55	3	0.32	3	3	3	0.161	4	
4	RPS7	0.56	4	0.27	1	2.99	2	0.223	8	
5	UbiQ	0.56	5	0.33	4	4.95	6	0.196	6	
6	RPL13-a	0.58	6	0.37	5	5.96	7	0.221	7	
7	RPL6	0.6	7	0.37	5	4.14	5	0.181	5	
8	V-ATPase	0.65	8	0.51	8	8.49	9	0.240	10	
9	ELF1	0.69	9	0.44	7	8.49	8	0.160	3	
10	Actin	0.74	10	0.7	11	10.72	10	0.269	11	
11	GADPH	0.74	11	0.6	10	10.74	11	0.295	12	
12	β-Tubulin	0.77	12	0.56	9	11.47	12	0.238	9	

**Table 3:** RNA-seq analysis results.

Description	R-Limonene vs. Control	Sabiene vs. Control
Number of total features	23,937	23,937
Number of filtered features	12,393	12,435
Number of features after filtering	11,544	11,502
DE (probability > 0.9)	5363	6815
Upregulated (M ≥ 1, probability > 0.9)	2069	2817
Downregulated (M ≤ −1, probability > 0.9)	1961	3761
Number of DE detoxification genes	294	426

**Table 4:** List of chemicals used in the bioassay with their purity.

Sr. No.	Name	Purity	Manufacturer	CAS
1	α-Pinene	97%	Thermo Scientific Chemicals, Waltham, MA, USA	80-56-8
2	(S)-(−)-Limonene	97%	Thermo Scientific Chemicals	5989-54-8
3	(R)-(+)-Limonene	~90%	Sigma-Aldrich, St. Louis, MO, USA	5989-27-5
4	(1S)-(+)-3-Carene	99%	Sigma-Aldrich	498-15-7
5	Myrcene	≥90%	Sigma-Aldrich	123-35-3
6	Sabiene	75%	Sigma-Aldrich	3387-41-5

**Table 5:** Primer designed for the RT-qPCR study.

Sr. No.	Primer Name	Transcript ID	Transcript Name		Primer Sequence	Amplicon Length	Tm (°C)
1	EST6	Ityp05867	Venom carboxylesterase-6	*F*	CAACCGAAATGGTGAACTG	119	60
				*R*	ATTCTCAACCACCGTAGAC		
2	HYEP1-a	Ityp10836	Juvenile hormone epoxide hydrolase 1	*F*	CGGCCTGACTAAACACTTT	110	60
				*R*	AGCCAAACCCTTCAGAATAC		
3	AK1A1	Ityp08881	Alcohol dehydrogenase	*F*	TCCGAACAACTGCAAAGG	160	60
				*R*	TAGCACCAGGACTTCCTAAA		
4	GST	Ityp13834	Glutathione S-transferase	*F*	CTACTCTGGAAGTGGATGG	138	58, 59
				*R*	AATCCGTGACTGTGTCG		
5	SDR1-a	Ityp00346	Farnesol dehydrogenase	*F*	GGCAATAACCACAAGAGAGG	139	60, 61
				*R*	CGCAAATTTACTGGCTGGA		
6	ABCGK	Ityp09976	ABC transporter G family member 20	*F*	CCACTGACGCTATTAAGCAC	143	61, 60
				*R*	CAGGTACGCCTTGATTTCTC		
7	IDLDH-a	Ityp22107	Ipsdienol dehydrogenase	*F*	GGACAATAATCGGGACGAAG	131	60
				*R*	GGTTGGTCATGGAGATGATG		
8	SDR1-b	Ityp13935	Farnesol dehydrogenase	*F*	GCGTTAACGGAAACTGTTAG	153	59
				*R*	AGGTCTGTCATTTGCTAGAG		
9	ST1B1	Ityp07436	Sulfotransferase 1B1	*F*	AACCACGTTCTGCCATTC	134	60
				*R*	GCTTCAGTGAGGTCTTTCTC		
10	CP6A2	Ityp03140	Cytochrome P450 6a2	*F*	TTGAGACATCGGCTACCA	156	60
				*R*	GGCGAAGATAGGTCATTTCC		
11	UDB17	Ityp14578	UDP-glucuronosyltransferase 2B17	*F*	GATTCCAACGCCGCTAAA	139	60
				*R*	GACGATGCTTCACTTGACTT		
12	HYEP1-b	Ityp03551	Juvenile hormone epoxide hydrolase 1	*F*	GAGAGATAGTCCGGT	127	60
				*R*	GTCCAGTAATTGGTC		
13	IDLDH-b	Ityp14704	Ipsdienol dehydrogenase	*F*	CAGACAGTTGGGACCTTTAG	146	60
				*R*	CACGTGGTTTGATCATTCTG		
14	SDHA	Ityp17457	Succinate dehydrogenase	*F*	CGTTCTGGATCTGTTGATGG	145	60
				*R*	GGCTGTGCAGGAGAAATATG		
15	GSTT1	Ityp05781	Glutathione S-transferase 1	*F*	GTAGATCAGCGCCTCCATTT	136	60
				*R*	GGGACTGATAAGCTTGCACCACT		
16	NCPR	Ityp11675	NADPH-cytochrome P450 reductase	*F*	GCAAACACTGCGAGAAGA	153	60
				*R*	AAACGTGAGTTCTGGGATTC		
17	DHB12	Ityp13083	17-beta-hydroxysteroid dehydrogenase 12	*F*	ATCAACAACGTCGGGATG	141	60
				*R*	CACCATTCCAGGAAGTACAA		
18	PA1B2	Ityp04924	Platelet-activating factor acetylhydrolase IB subunit beta homolog	*F*	ACTACCTCGAGGACAGAATC	136	60
				*R*	ACCATCAGGTTGGATAAAGC		
19	DHGL	Ityp11581	Glucose dehydrogenase	*F*	GGCTTTCAGAAGTGGAGAAT	140	60
				*R*	GTTCTGGACGGTGGTATTG		

## Data Availability

The original contributions presented in this study are included in the article; further inquiries can be directed to the corresponding author. The RNA-seq raw reads were submitted under NCBI bioproject PRJNA1149972.

## Supplementary Materials

The following supporting information can be downloaded at: https://www.mdpi.com/article/10.3390/ijms251810209/s1. Reference [109] is cited in Supplementary File S2.
