# Supplementary material for: Insights into the Detoxification of Spruce Monoterpenes by the Eurasian Spruce Bark Beetle
Source: Int J Mol Sci. 2024 Sep 23;25(18):10209. doi: 10.3390/ijms251810209 (PMC11432361; doi:10.3390/ijms251810209)
Supplement: Supplementary file 1 [file ijms-25-10209-s001.zip › S2_Analysis_report.pdf]

# Pairwise Differential Expression Results

Name: L1_vs_C1

## Dataset Overview

- Number of total features: **23,937**
- Number of filtered features: **12,393**
- Number of features after filtering: **11,544**
- Number of analyzed samples: **2**

## Results

Number of differentially expressed (DE) features (Probability > 0.9): **5,363**

- Up-regulated (M > 0): **2,655**
- Down-regulated (M < 0): **2,708**

## Experimental Design

| Sample | Raw lib. size | Normalized lib. size | Condition |
|---|---|---|---|
| C1 | 19,241,190 | 24,332,773.402 | Reference |
| L1 | 35,242,306 | 30,454,310.168 | Contrast |

## Analysis Parameters

| Parameter | Value |
|---|---|
| CPM Filter | 1.0 |
| Normalization Method | TMM (Trimmed mean of M values) |
| Number of Simulated Replicates | 5 |
| Size of the Simulated Replicates | 0.2 |
| Variability | 0.02 |
| Contrast Condition | L1 |
| Reference Condition | C1 |

## References


- Tarazona S., Furio-Tari P., Turra D., Pietro AD., Nueda MJ., Ferrer A. and Conesa A. (2015). Data quality aware analysis of differential expression in RNA-seq with NOISeq R/Bioc package. *Nucleic acids research, 43*(21), e140.
- Tarazona S., Garcia-Alcalde F., Dopazo J., Ferrer A. and Conesa A. (2011). Differential expression in RNA-seq: a matter of depth. *Genome research, 21*(12), 2213-23.
- OmicsBox - Bioinformatics made easy. BioBam Bioinformatics (Version 3.2.2). March 3, 2019. www.biobam.com/omicsbox.

# Pairwise Differential Expression Results

Name: S1_vs_C1

## Dataset Overview

- Number of total features: **23,937**
- Number of filtered features: **12,435**
- Number of features after filtering: **11,502**
- Number of analyzed samples: **2**

## Results

Number of differentially expressed (DE) features (Probability > 0.9): **6,815**

- Up-regulated (M > 0): **2,969**
- Down-regulated (M < 0): **3,846**

## Experimental Design

| Sample | Raw lib. size | Normalized lib. size | Condition |
|---|---|---|---|
| C1 | 19,241,190 | 15,257,786.876 | Reference |
| S1 | 17,282,404 | 21,832,888.499 | Contrast |

## Analysis Parameters

| Parameter | Value |
|---|---|
| CPM Filter | 1.0 |
| Normalization Method | TMM (Trimmed mean of M values) |
| Number of Simulated Replicates | 5 |
| Size of the Simulated Replicates | 0.2 |
| Variability | 0.02 |
| Contrast Condition | S1 |
| Reference Condition | C1 |

## References


- Tarazona S., Furio-Tari P., Turra D., Pietro AD., Nueda MJ., Ferrer A. and Conesa A. (2015). Data quality aware analysis of differential expression in RNA-seq with NOISeq R/Bioc package. *Nucleic acids research, 43*(21), e140.
- Tarazona S., Garcia-Alcalde F., Dopazo J., Ferrer A. and Conesa A. (2011). Differential expression in RNA-seq: a matter of depth. *Genome research, 21*(12), 2213-23.
- OmicsBox - Bioinformatics made easy. BioBam Bioinformatics (Version 3.2.2). March 3, 2019. www.biobam.com/omicsbox.
